# Decreased biofilm formation ability of *Acinetobacter baumannii* after spaceflight on China's Shenzhou 11 spacecraft

**DOI:** 10.1002/mbo3.763

**Published:** 2018-10-31

**Authors:** Xian Zhao, Yi Yu, Xuelin Zhang, Bing Huang, Po Bai, Chou Xu, Diangeng Li, Bin Zhang, Changting Liu

**Affiliations:** ^1^ Nanlou Respiratory Diseases Department Chinese PLA General Hospital Beijing China

**Keywords:** *Acinetobacter baumannii*, genome, phenotype, spaceflight, transcriptome

## Abstract

China has prepared for construction of a space station by the early 2020s. The mission will require astronauts to stay on the space station for at least 180 days. Microbes isolated from the International Space Station (ISS) have shown profound resistance to clinical antibiotics and environmental stresses. Previous studies have demonstrated that the space environment could affect microbial survival, growth, virulence, biofilms, metabolism, as well as their antibiotic‐resistant phenotypes. Furthermore, several studies have reported that astronauts experience a decline in their immunity during long‐duration spaceflights. Monitoring microbiomes in the ISS or the spacecraft will be beneficial for the prevention of infection among the astronauts during spaceflight. The development of a manned space program worldwide not only provides an opportunity to investigate the impact of this extreme environment on opportunistic pathogenic microbes, but also offers a unique platform to detect mutations in pathogenic bacteria. Various microorganisms have been carried on a spacecraft for academic purposes. *Acinetobacter baumannii* is a common multidrug‐resistant bacterium often prevalent in hospitals. Variations in the ability to cope with environmental hazards increase the chances of microbial survival. Our study aimed to compare phenotypic variations and analyze genomic and transcriptomic variations in *A. baumannii* among three different groups: SS1 (33 days on the Shenzhou 11 spacecraft), GS1 (ground control), and Aba (reference strain). Consequently, the biofilm formation ability of the SS1 strain decreased after 33 days of spaceflight. Furthermore, high‐throughput sequencing revealed that some differentially expressed genes were downregulated in the SS1 strain compared with those in the GS1 strain. In conclusion, this present study provides insights into the environmental adaptation of *A. baumannii* and might be useful for understanding changes in the opportunistic pathogenic microbes on our spacecraft and on China's future ISS.

## 1 INTRODUCTION

Space is an extremely complex environment in which extreme conditions, including microgravity, low temperature, strong radiation, and nutrient‐deprivation, are encountered. Certain microbes can survive under these conditions. Mora et al. (2016) analyzed dust samples obtained from inside the ISS. After culturing, 85 bacterial isolates were identified. Most of these isolates showed profound resistance to clinical antibiotics and environmental stresses in the ISS environment; moreover, they did not encounter UV radiation. Ilyin (2005) collected microbial samples from astronauts of the Salyut and Mir space programs after short and long spaceflight missions. The population of opportunistic pathogens reportedly increased, accompanied by changes in antibiotic resistance during spaceflight. The spaceflight condition affected both the internal and external microflora in humans (Liu, 2017). The immune system of the astronaut during spaceflight was reportedly negatively altered during spaceflight. Production of interleukin (IL)‐2 and interferon (IFN) γ from T cells decreased. Furthermore, immunoglobulins (e.g., IgG and IgM) and the cytotoxic properties of natural killer (NK) cells also reportedly decreased (Crucian, Stowe, Pierson, & Sams, 2008; Ilyin, 2005). The study by Mehta et al. (2014) provided more evidence of astronauts exhibiting low immunity after spaceflight. In their study that involved 17 astronauts, they found that Epstein–Barr virus, varicella‐zoster virus, and cytomegalovirus were reactivated during spaceflight. The risk of infection increased during spaceflight. These changes not only affect astronauts’ health during spaceflight, but also influence life on Earth because microbes that accompany astronauts into space eventually return to earth with the spacecraft. The National Aeronautics and Space Administration, European Space Administration, and Japan Aerospace Exploration Agency have initiated missions to elucidate the adaptation of microorganisms to the microgravity environment and the molecular mechanisms underlying the effects of microgravity on microbes and humans (Yamaguchi et al., 2014). The MARS 500 study by Schwendner et al. (2017) provided an important estimation of the potential risks astronauts encounter during spaceflight, providing essential information regarding spacecraft design, microbial monitoring strategies, and microbiological environmental safety during spaceflight. Monitoring phenotypic changes in microorganisms during spaceflight may be beneficial to prevent and cure infection (Liu, 2017).

Studies on microbial changes and adaptations during spaceflight have been reported; some opportunistic bacterial or fungal pathogens were carried in the spacecraft or cultured in the ISS. Coil et al. (2016) collected from built environment and cultured *Bacillus safensis* both in the ISS and on ground, and reported that *B. safensis* growth was markedly better in space than on ground. Nicholson et al. (2011) reported changes in growth rates of *Bacillus subtilis* spores carried by the O/OREOS spacecraft. Crabbé et al. (2013) reported enhanced cell invasion and binding in *Candida albicans* cultured during spaceflight compared to those on ground. *Escherichia coli* and *B. subtilis* cultured in space shuttle mission STS‐63 displayed a greater density of cells in the stationary phase, with a shorter lag‐phase (Kacena, Merrell, et al., 1999). Drug resistance of *Staphylococcus epidermidis*,* Klebsiella pneumoniae*, and *Bacillus cereus* was reportedly altered (Fajardo‐Cavazos, 2018; Fajardo‐Cavazos & Nicholson, 2016; Guo et al., 2014; Su et al., 2014), and drug resistance persisted in *K. pneumoniae* and *B. subtilis* cultured in a spacecraft or in the ISS (Guo et al., 2015; Morrison et al., 2017). Previous studies have reported an increased incidence of mutations in bacteria after spaceflight (Sulzman & Genin, 1994). Additionally, in the microgravity environment, survival, growth, virulence, and antibiotic resistance phenotypes have been shown to be mutated (Horneck, Klaus, & Mancinelli, 2010; Rosenzweig et al., 2010). Studies on genomic changes as a function of bacterial adaptation to a special environment, such as space, could reveal the mechanisms underlying microbial adaptation and responses to extreme environments.

China has launched the orbiting space laboratory Tiangong 2 to prepare for the construction of a space station by the early 2020s. The mission will require astronauts to stay on the space station for at least 180 days. China has also launched Shenzhou I, Shenzhou III, and Shenzhou IV spacecraft in 1999–2002, and for academic purposes, *Streptomyces fradiae* was harbored on these spacecraft (Fang, Zhao, & Gu, 2005). A few years later, 15 strains of microorganisms were harbored on the Shenzhou VIII spacecraft, and 2 years later, the Shenzhou X spacecraft, which carried nine different microorganisms, was launched. Phenotypic, genomic, and transcriptomic changes were detected in these microorganisms, including drug resistance mutations and metabolic adaptations to the space environment (Chang et al., 2013; Guo et al., 2014; Li et al., 2014; Su, Chang, & Liu, 2013; Su et al., 2014; Zhang, Fang, & Liu, 2015).

The spaceflight environment not only aids in studying the influence of spaceflight on bacteria to evaluate the risk of infection among astronauts, but also helps to develop potential vaccine targets and therapeutic studies against persistent infections caused by pathogenic bacteria (Higginson, Galen, Levine, & Tennant, 2016). *Acinetobacter baumannii*, a common opportunistic pathogen isolated from paleosol, displayed 87% similarity to that recovered from a clinical environment (Hrenovic, Durn, Goicbarisic, & Kovacic, 2014). *Acinetobacter baumannii* infections often occur in patients with low immunity, and this microbe displays robust resistance to most clinical antibiotics. The virulence and drug resistance mechanisms of *A. baumannii* reportedly include resistance to disinfectants, desiccation, oxidative stress, biofilm formation, and glycoconjugates (Harding, Hennon, & Feldman, 2017). In this study, we aimed to investigate phenotypic changes in *A. baumannii* during spaceflight and determine the mechanisms underlying their adaptation to the space environment and changes in virulence via genetic and transcriptomic analyses. *Acinetobacter baumannii* cells were cultured onboard the Shenzhou 11 spacecraft with matched controls on Earth. Our findings may provide insights into the changes in bacteria on our spacecraft, and on China's future ISS (Kass, 1971; Rosenzweig et al., 2010).

## 2 MATERIALS AND METHODS

### 2.1 Bacterial strains and culture conditions

The original *A. baumannii* strain used in the study was isolated from a sputum sample of a patient with pneumonia at the Nanlou Respiratory Department of the Chinese PLA General Hospital. This strain—designated “Aba”—was stored at −80˚C and was used as a baseline reference. *Acinetobacter baumannii* was inoculated into a plastic container filled with semisolid Luria‐Bertani (LB) medium (with 0.5% agar), under the following two conditions: (a) the SS1 sample was first transported to the China Astronaut Research and Training Center within 30 min, and then to Jiuquan from Beijing within 6 hr by military plane, followed by on‐boarding the Shenzhou 11 spacecraft (launched on 17 October 2016, and landed on 18 November 2016) and (b) the SS1 strain was transferred from Siziwangqi, where the cabin landed. Six hours later, SS1 was transferred to the laboratory in Beijing by military plane. The GS1 strain was cultured on ground for 33 days at the same temperature as that for the SS1 strain in the Shenzhou 11 spacecraft at 21°C.

### 2.2 Disk diffusion test

This assay was performed to test the susceptibility of Aba, SS1, and GS1 strains. The following drugs were used in accordance with the CLSI M100‐S24 document (CLSI, 2014): ceftazidime, imipenem, ceftriaxone, aztreonam, cefepime, ciprofloxacin, tobramycin, ampicillin/sulbactam, trimethoprim‐sulfamethoxazole, amikacin, meropenem, and levofloxacin. The entire surface of the MH agar plate was seeded with bacteria. The volume of the bacterial suspension was 1.5 ml, with the density of each strain adjusted to 10^7^–10^8^ CFU/ml. Disks were placed on the surface of the MH plate. The diameter of the zone of inhibition was measured after culturing at 37°C for 18 hr. All disk diffusion tests for the three strains were performed in thrice.

### 2.3 Scanning electron microscopy (SEM)

Aba, GS1, and SS1 strains were cultured in LB medium, washed with sterile phosphate‐buffered saline (PBS; pH 7.4), and fixed with 4% glutaraldehyde overnight. The samples were washed again with PBS, dehydrated in increasing grades of ethanol, and then critical point‐dried. After coating the specimens with gold‐palladium, they were observed using an FEI Quanta 200 scanning electron microscope (USA).

### 2.4 Growth curves

SS1, GS1, and Aba strains were cultured in 15 ml LB medium at 37°C for 24 hr with agitation. The optical density at 630 nm was measured using 200 μl of SS1, GS1, and Aba strains every 2 hr for 24 hr.

### 2.5 Analysis of biofilm formation ability via crystal violet staining

Two‐hundred microliters of the bacterial suspension culture (1–5 × 10^7^ CFU/ml) was added to each well of a 96‐well flat‐bottom microtiter plate and cultured at 37°C for 24 hr. The wells were then washed twice with PBS, and cells were stained with 200 μl of 0.1% crystal violet (Sigma, St. Louis, MO, USA) for 30 min. The plates containing the three strains were washed with PBS. After drying, the stained biomass samples were dissolved in 95% ethanol. The OD_540_ for each well was determined using a Thermo Multiskan Ascent instrument (Thermo, USA). All experiments were performed in triplicate.

### 2.6 Analysis of biofilm formation ability via confocal laser‐scanning microscopy (CLSM)

The three strains were cultured on MH agar plates overnight and inoculated in 35‐mm confocal dishes (Solarbio, Beijing, China). The plates were incubated at 37°C for 24 hr and stained with a Filmtracer LIVE/DEAD Biofilm Viability Kit (Invitrogen, Carlsbad, CA, USA) in accordance with the manufacturer's instructions. Three image stacks were obtained from each sample randomly by CLSM using a Leica TCS SP2 microscope with a Lecia TCS SP2/AOBS imaging system.

### 2.7 Genome sequencing

Genomic DNA from Aba, SS1, and GS1 strains was obtained using the sodium dodecyl sulfate extraction method (Ausubel, 2002). After electrophoretic detection, DNA samples from the three strains were quantified using Qubit fluorometer. Library construction and sequencing were performed at the Beijing Novogene Bioinformatics Technology.

Thereafter, for the Aba DNA sample, a 10‐kb SMRT Bell library was constructed in accordance with standard quality controls. Sequencing was performed using a PacBio RSII. Low‐quality reads of the original strain were filtered using SMRT 2.3.0 (Berlin et al., 2015; Koren & Phillippy, 2015). The filtered reads were then assembled to generate one contig without gaps. We used GeneMarkS to predict genome components. Gene functions were then predicted using gene databases related to function, pathogen, and virulence databases.

The genomes of strains SS1 and GS1 were sequenced using high‐throughput sequencing Illumina technology. A 350‐bp library was constructed and sequenced using an Illumina HiSeq4000 platform, using the PE150 strategy. An in‐house program was used for quality control of the paired‐end reads. The original data obtained using the Illumina HiSeq4000 platform were filtered by removing reads that contained adapters and poly‐N, as well as low‐quality reads from the raw data. Clean data were used to map the reads.

The original Aba strain was used as a reference. The SS1 and GS1 reads were mapped to the reference sequence using BWA (version: 0.7.8) (Li Heng and Durbin (2009), and the coverage was determined using SAMTOOLS (version: 0.1.18; Li Heng et al., 2009). SAMTOOLS (version: 0.1.18) was used to detect single‐nucleotide polymorphisms (SNPs) and insertion/deletion (InDel) mutations. Sequence variation (SV) analysis, including insertions (INSs), deletions (DELs), inversions (INVs), intrachromosomal translocation (ITXs), and interchromosomal translocations (CTXs), was performed using BreakDancer (version 1.4.4) (Chen et al., 2009).

### 2.8 RNA sequence and comparative transcriptomic data analysis

RNA was isolated immediately from the cells incubated in semisolid LB medium. Total RNA samples of the three strains were obtained using an RNeasy Protect Bacteria Mini Kit (Qiagen, Germany) following the product instruction. Three micrograms of RNA from each sample was used for RNA sample preparation. After assessing the quality of the RNA samples, sequencing libraries were constructed using a NEBNext Ultra Directional RNA Library Prep Kit for Illumina (NEB, USA) in accordance with the manufacturer's instructions. Thereafter, initial quantitative analysis was performed using Qubit2.0. The library was diluted to 0.1 ng/μl. After assessing DNA quality using an Agilent 2100 system, quantitative polymerase chain reaction was performed for accurate quantification. After cluster generation, the constructed library was sequenced on an Illumina HiSeqTM2500 platform. Clean reads were obtained by deleting reads containing low‐quality reads, reads with adapters, and reads containing poly‐*N* (>10%) from the raw data. Bowtie2‐2.2.3 (Langmead & Salzberg, 2012) was used to locate clean reads to the reference genome. HTSeq v0.6.1 was used to quantify gene expression, and the fragments per kilobase of transcript per million mapped reads (FPKM) of each gene was calculated in accordance with gene length. Finally, read counts were mapped to the genes.

Differential gene expression analysis of the three strains was performed using the DESeq R package (1.18.0). Genes yielding a *p* value of <0.05 upon DESeq analysis were considered differentially expressed. Gene ontology (GO) enrichment analysis of differentially expressed genes (DEGs) was carried out using the GOseq R package. GO terms with *p* values of <0.05 were considered significantly enriched by DEGs. KOBAS software was used to assess the meaningful statistical enrichment of DEGs in Kyoto Encyclopedia of Genes and Genomes (KEGG) pathways.

### 2.9 Statistical analysis

Differences in growth curves and biofilm formation abilities among the Aba, SS1, and GS1 strains were evaluated using one‐way analysis of variance, followed by Tukey's multiple comparison test for post hoc analysis. GraphPad Prism (version 7.00) was used for data analysis. Differences with *p* values of <0.05 were considered significant.

## 3 RESULTS

### 3.1 Phenotypic characteristics of *Acinetobacter baumannii* strains

The results of the disk diffusion assays revealed that the Aba and GS1 strains were resistant to all tested antibiotic disks. Notably, this drug resistance characteristic of the SS1 strain remained unchanged after spaceflight (Table 1).

**Table 1:** Antibiotic sensitivity analysis of the three strains used in this study

Antibiotics	Inhibition zone diameter (mm)		
	Aba	GS1	SS1
Ceftazidime	0/R	0/R	0/R
Imipenem	5/R	5.8/R	5.4/R
Ceftriaxone	0/R	0/R	0/R
Aztreonam	9.4/R	8.2/R	7.6/R
Cefepime	5/R	5/R	4.4/R
Ciprofloxacin	0/R	0/R	0/R
Tobramycin	12/R	12/R	12/R
Ampicillin/sulbactam	6.2/R	6.8/R	6.8/R
Trimethoprim‐sulfamethoxazole	10/R	10/R	10/R
Amikacin	14.4/R	14.8/R	14.8/R
Meropenem	5/R	6/R	6/R
Levofloxacin	7/R	5.8/R	5.8/R

**Figure 1:**
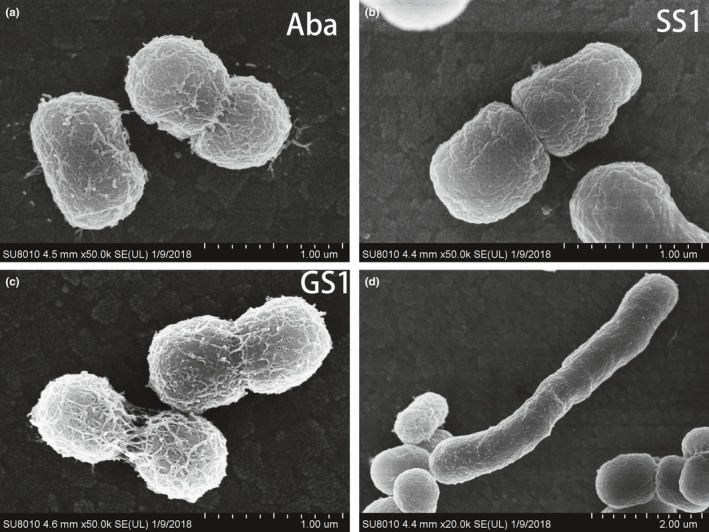
Scanning electron micrographs (SEM) of Aba (A), SS1 (B/D), and GS1 (C) strains. (A) SEM of the Aba strain. (B)The flight strain SS1 showed the least intercellular mucus and the smoothest cell wall. (C) SEM of the GS1 strain. (D) Some elongated cells were observed for the SS1 strain upon SEM imaging

**Figure 2:**
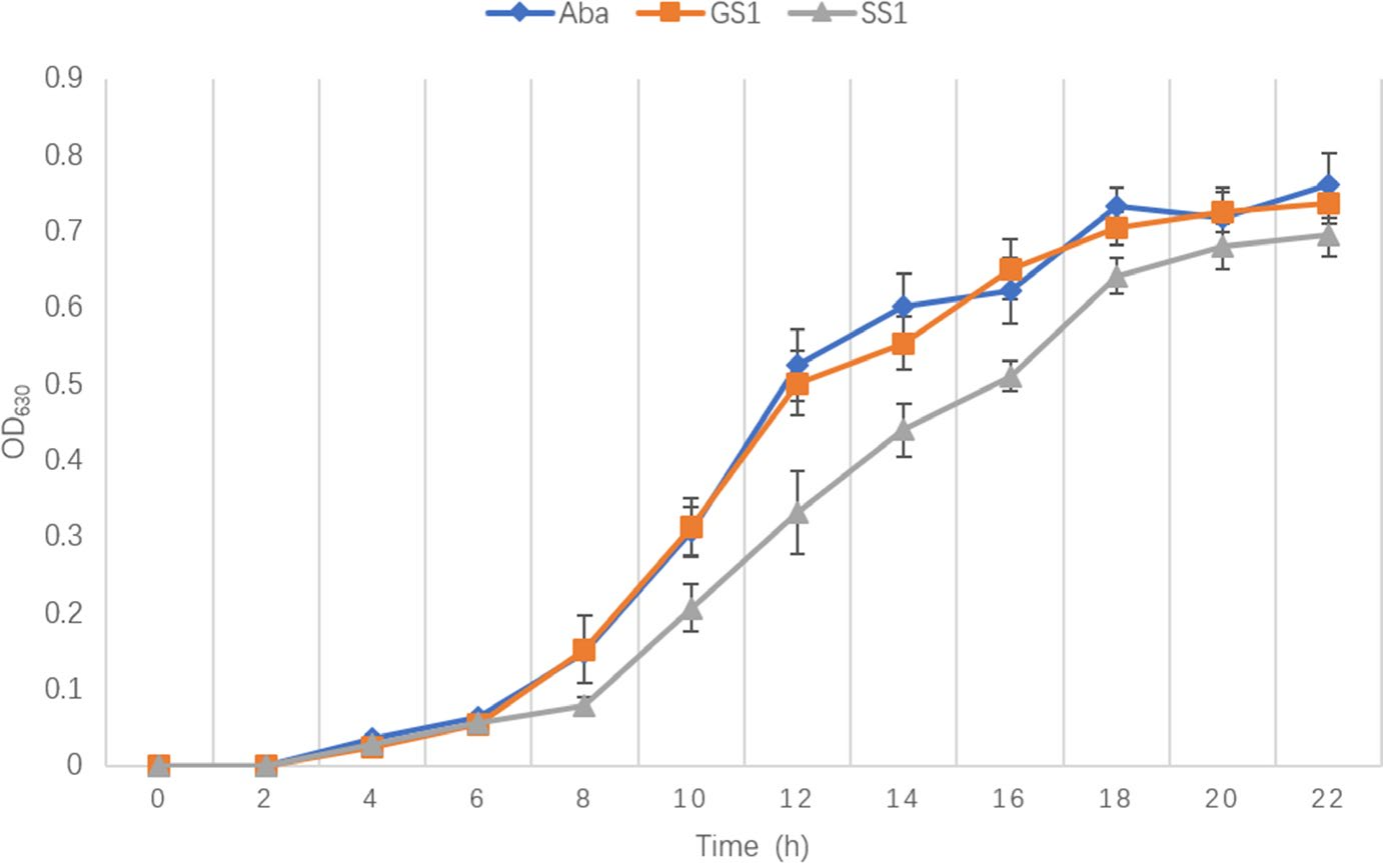
Growth rates of the Aba, SS1, and GS1 strains. *p* was 0.0315 at 8 hr. SS1, GS1, and Aba strains were cultured in 15 ml LB medium at 37°C for 24 hr with agitation. The optical density at 630 nm was measured using 200 μl of SS1, GS1, and Aba strains every 2 for 24 hr. The experiment was repeated three times

Scanning electron microscopy was then performed to observe single cell morphology in the Aba, SS1, and GS1 strains. The results showed that the flight strain SS1 had less intercellular mucus and smoother cell walls than the GS1 and Aba strains. This may have decreased its biofilm formation ability. Furthermore, some elongated cells in the SS1 strain were observed upon SEM analysis (Figure 1). The SS1 and GS1 strains also displayed differences in their growth curves. Growth was significantly slower in the SS1 strain than in the GS1 strain, particularly after 8 hr (*p = *0.0315). Similar growth rates were observed in GS1 and Aba strains (Figure 2).

**Figure 3:**
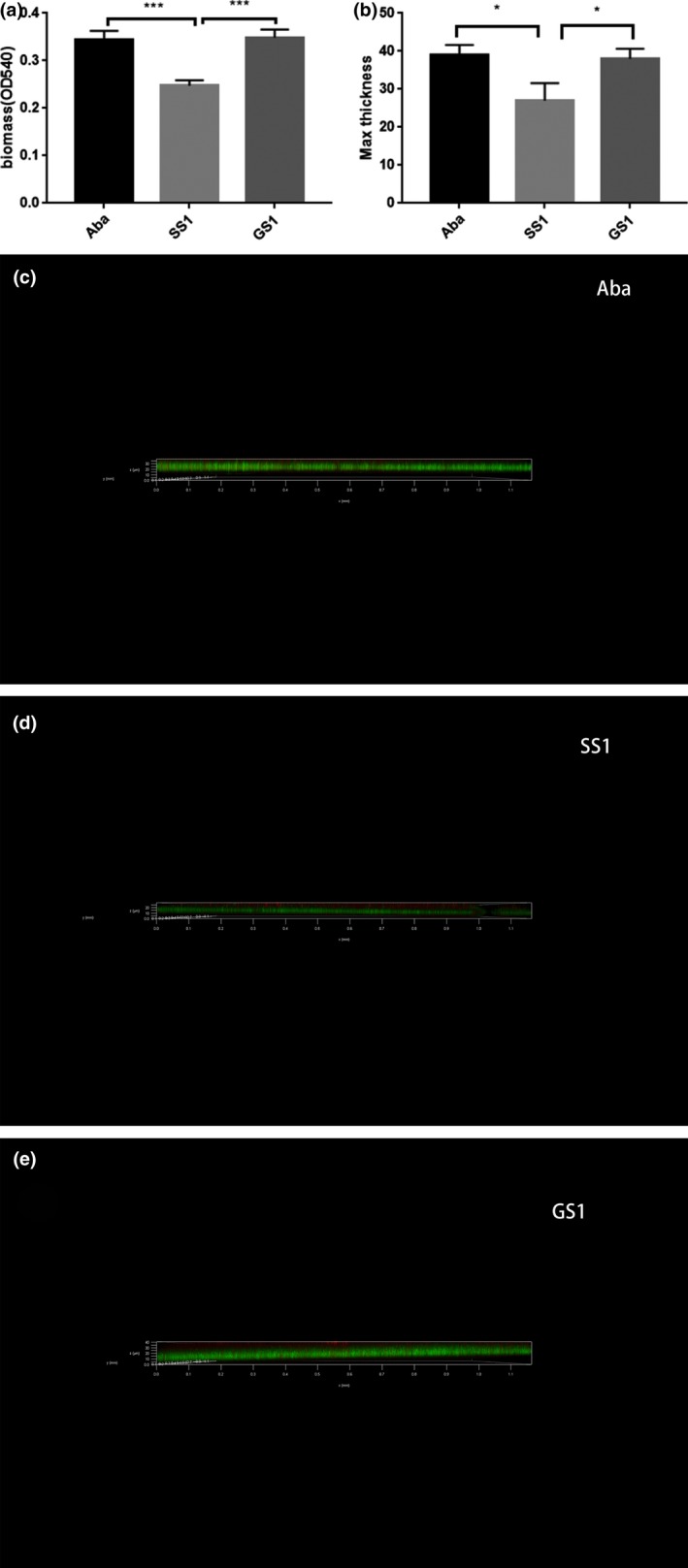
(a)Analysis of biofilm formation ability via Crystal Violet staining. The three strains were cultured in 96‐well polystyrene microtiter plates at 37°C for 24 hr. Biofilm formation ability was measured by determining the optical density at 540 nm. (b) Biofilms of SS1, GS1, and Aba strains cultured in 35‐mm confocal dishes. Cells were stained with a Filmtracer LIVE/DEAD Biofilm Viability Kit, and biofilm formation ability was tested using confocal laser‐scanning microscopy (CLSM). Green fluorescence indicates live cells; red fluorescence indicates dead cells. ***adjusted *p* value <0.01; *adjusted *p* value <0.05. (c) Analysis of biofilm formation ability of Aba strain using CLSM. (d) Analysis of biofilm formation ability of SS1 strain using CLSM. (e) Analysis of biofilm formation ability of GS1 strain using CLSM

The biofilm formation ability of *A. baumannii* is an important characteristic of environmental adaptability and virulence and is related to adhesion, antibiotic resistance, and reduced immune recognition. Therefore, we performed crystal violet staining to assess biomass of the Aba, SS1, and GS1 strains. The OD_540_ of SS1 was significantly lower than that of GS1 (*p* = 0.0006). The biofilm formation ability of the SS1 strain was lower than that of the GS1 strain, while the biofilm formation ability of the GS1 strain was similar to that of the reference strain (Figure 3). Importantly, these results were further confirmed via CLSM analysis. The three image stacks obtained from each strain showed that the maximum biofilm thickness in SS1 was significantly less than that in both GS1 (*p* = 0.0178) and Aba (*p* = 0.0120).

### 3.2 Overview of the Aba strain

**Figure 4:**
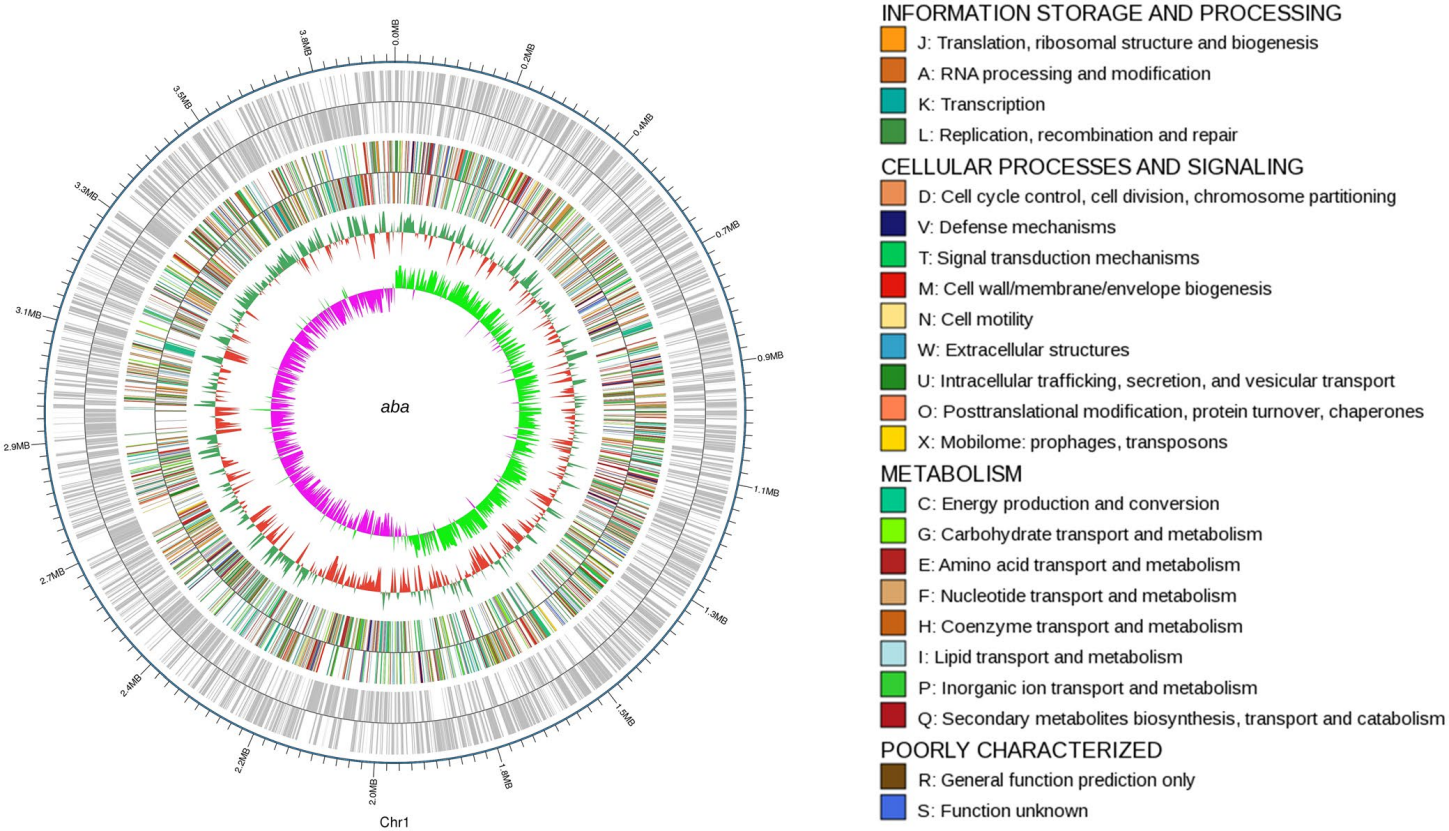
Circular representation of the genomic features of the reference strain. From the outer circle to the inner ring: genome sequences, cluster of orthologous groups annotations, GC content, and GC skew

The accession number of the Aba strain is CP030083–CP030084. The draft genome of the Aba strain was estimated to be of 3.91 Mbp and to have a GC content of 39.04%. Additionally, this strain was predicted to have 3,722 coding sequences, 73 tRNAs, one sRNA, and 12 gene islands. The average length of the coding sequences was 924 bp, encompassing 87.68% of the genome. More genome features are shown in the circular representation in Figure 4.

### 3.3 Genome comparisons

**Figure 5:**
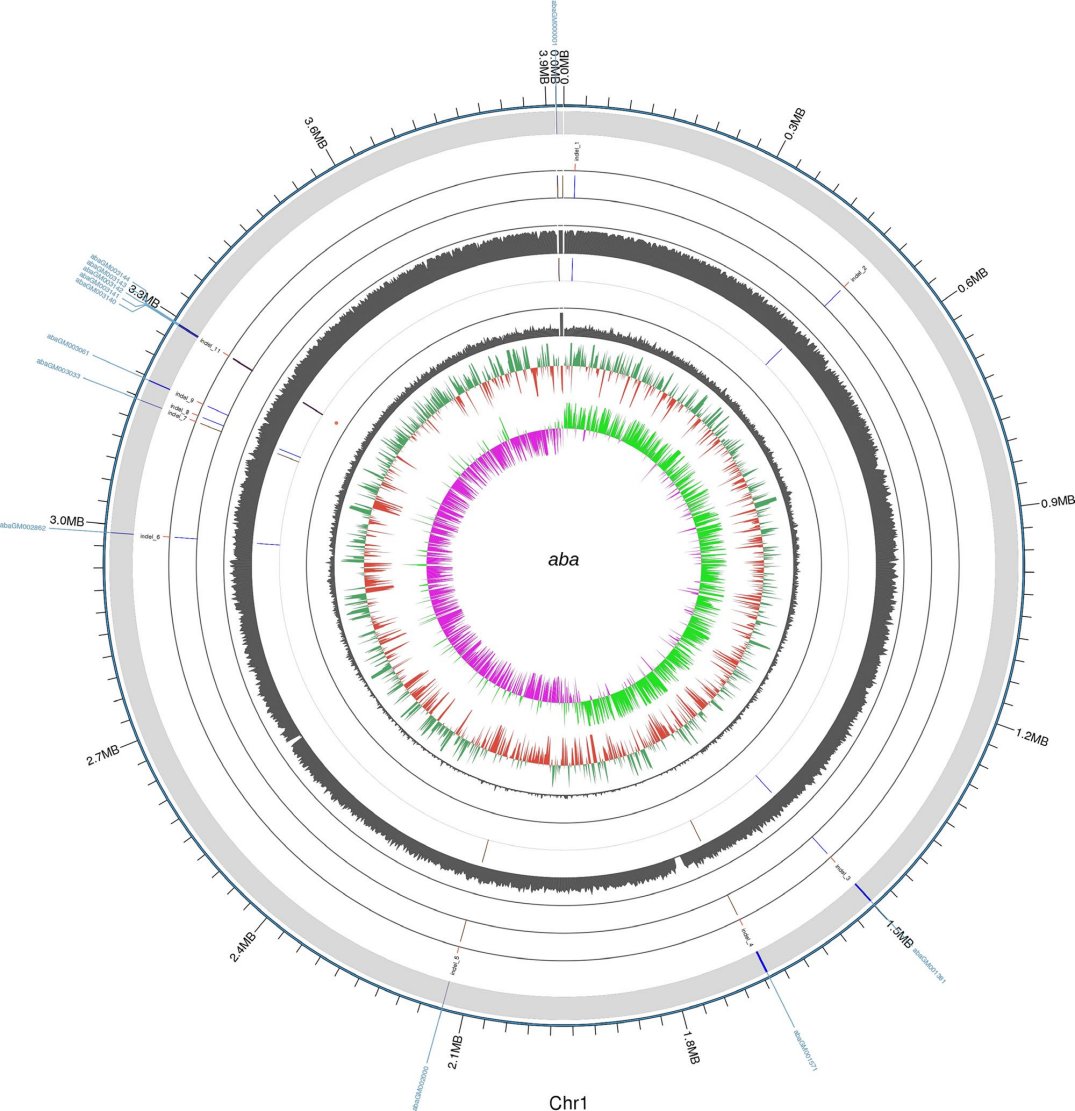
Whole‐genome mutation profile. From the outer circle to the inner ring: InDels, read coverage of the GS1 strain; InDels, SNPs, and read coverage of the SS1 strain. The insertions are indicated in yellow; deletions, blue

The accession number for resequencing data of the SS1 strain is SAMN09453122; GS1 strain, SAMN09453123. In total, 8,772,440,845 bases from the SS1 and GS1 strains were mapped to the Aba genome to estimate genomic changes during spaceflight. Only one SNP was identified in the SS1 strain, and this SNP was a nonsynonymous mutation detected in the predicted coding sequence. Identification of the mutations in the SS1 and GS1 strains compared with the Aba strain identified eight and seven insertions and 12 and 11 deletions in GS1 and SS1, respectively. The gene abaGM003061 was only identified in the GS1 strain (Figure 5).

### 3.4 Comparative transcriptomic analysis

**Figure 6:**
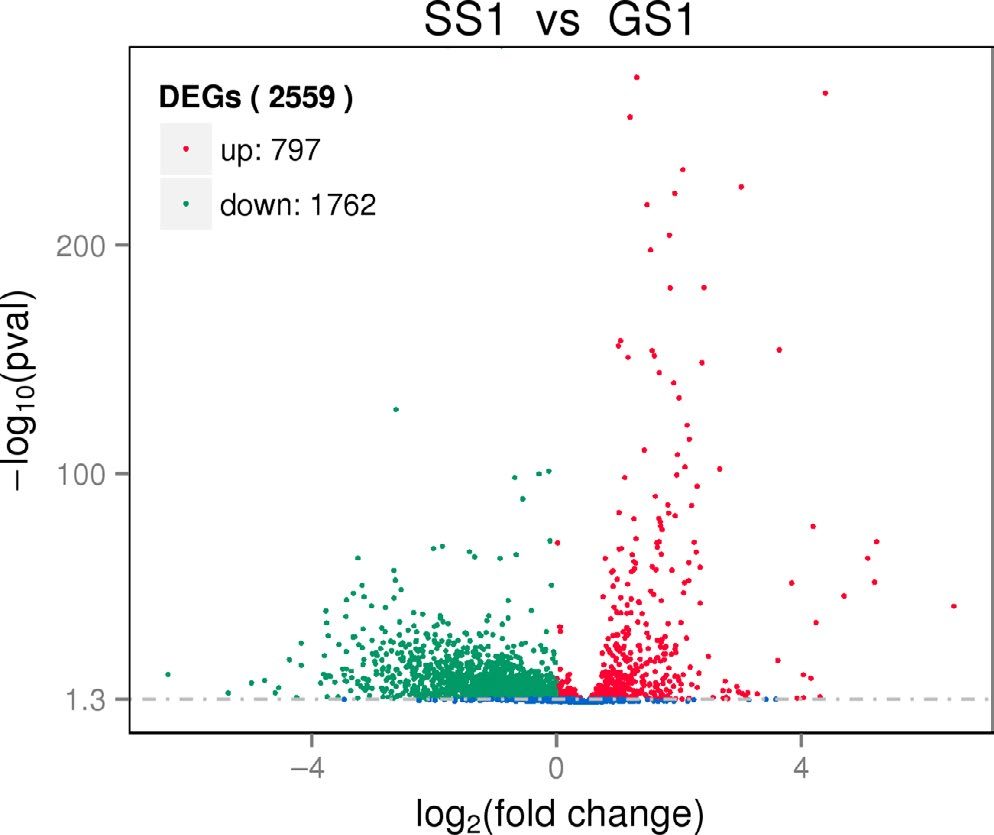
Upregulated and downregulated genes in the SS1 strain compared with genes expressed in the GS1 strain

**Figure 7:**
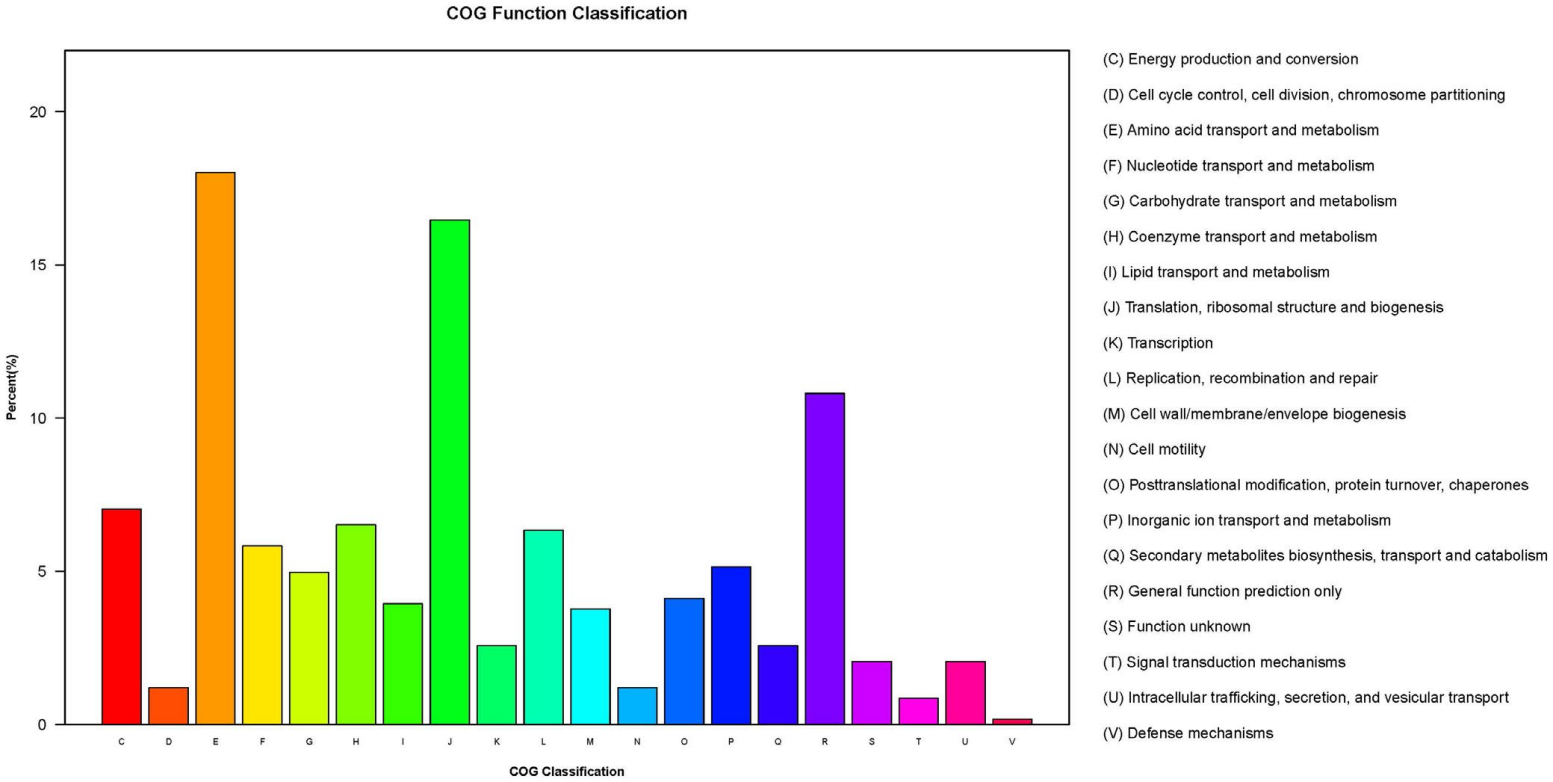
Distribution of differentially expressed genes (DEGs) between SS1 and GS1 in cluster of orthologous group functional categories. 583 DEGs were mapped to 20 COG categories, 18% of which were related to amino acid transport and metabolism. 16.5% were related to translation, ribosomal structure, and biogenesis

The accession number for transcriptomic data of the SS1 strain is SAMN09453347; GS1 strain, SAMN09453348. Overall, 2,559 DEGs were defined between SS1 and GS1 depending on the FPKM value. In total, 797 genes were upregulated and 1,762 were downregulated (Figure 6). Additionally, 583 DEGs were mapped to cluster of orthologous group (COG) categories (Figure 7), most of which are associated with ribosomal structure and biogenesis, amino acid transport, and metabolism and translation. There were no DEGs in the extracellular structure mobilome or in the prophage, transposon, and RNA processing and modification categories (Figure 6). The *csuA*/*B*/*D* genes, which are associated with biofilm formation, were downregulated.

**Figure 8:**
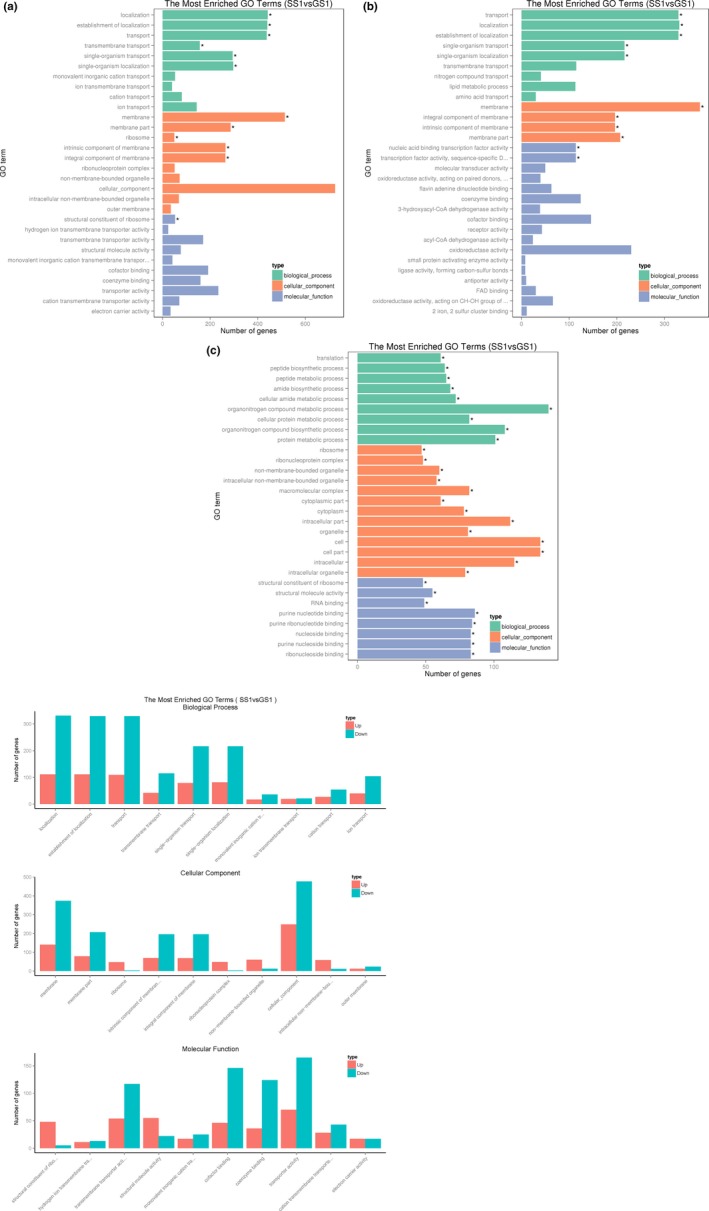
(a) Distribution of differentially expressed genes (DEGs) in gene ontology (GO) functional categories. (b) Distribution of DEGs downregulated in GO functional categories. (c) Distribution of DEGs upregulated in GO functional categories. (d) Distribution of DEGs up/down in GO functional categories. Green means downregulated, and red means upregulated

Differentially expressed genes were also mapped to GO categories (Figure 8). DEGs were enriched in cellular components, molecular functions, and biological processes. For molecular functions, DEGs between SS1 and GS1 were classified into 26 categories. For biological processes, DEGs were classified into 19 meaningful categories; cellular components, 14 meaningful categories; cellular component, 14 categories, including 2,511 DEGs, were meaningful (note that the same DEG may exist in different categories). In the cellular component category, some categories were associated with the formation of the membrane, including the membrane (*p* = 1.8e−05), membrane part (*p = *3.86e−05), and intrinsic membrane components (*p = *1.40e−04). In the biological process category, 19 categories, including localization (*p* = 0.000035195), establishment of localization (*p* = 4.63e−05), and transport (*p* = 5.79e−05), including 2,543 DEGs, were meaningful. These 19 categories were related to location and transport. Most DEGs enriched in these categories in SS1 and GS1 were downregulated. In the molecular function category, 26 categories, such as transporter activity (*p = *1.11e‐02), cofactor binding (*p = *6.37e‐03), and transmembrane transporter activity (*p = *2.79e‐03), including 2,110 DEGs, were meaningful. Most of these DEGs were also downregulated. Numerous biological activities, including metabolism and transmembrane transporter, were decreased in SS1.

Furthermore, only one KEGG pathway (K02014, *p* = 0.029; related to iron complex outer membrane receptor protein) comprising 20 DEGs was particularly meaningful, and 18 genes in this pathway were downregulated.

Using the virulence factors of pathogenic bacteria (VFDB)‐based annotation system to identify virulence factors, we identified 50 genes displaying transcriptomic changes in the SS1 strain in comparison with the GS1 strain. Thirteen genes were upregulated and 34 were downregulated. Nine of the downregulated genes were associated with virulence factors and type IV pili, which are associated with biofilm formation, including the *pilC* (*p* 1.48e−10) and *pilJ* (*p* 1.41e−7) genes. Two downregulated genes, *algB* (*p* 1.97e−04) and *algR*, were also associated with the virulence factor alginate, which contributes to the biofilm formation ability. Finally, the *fim3* and *fimB* genes, which are associated with the virulence factor fimbriae, were also downregulated.

Using the Antibiotic Resistance Genes Database‐based annotation system, we identified four genes showing transcriptomic changes in the SS1 strain compared with the GS1 strain. Briefly, the *Mdfa* (*p* 1.83e−19), *acr* (*p* 0.014459), *bla_a* (*p* 5.49e−20), and *aph* (*p* 2.00e−03) genes were upregulated.

Finally, using the Pathogen‐Host Interactions database‐based annotation system, we identified 51 genes showing transcriptomic changes in the SS1 strain. Of these genes, 33 were related to reducing virulence, and 19 of the DEGs were upregulated.

## 4 DISCUSSION

Some of the bacteria found in the ISS were opportunistic pathogens and were resistant to stress and antibiotics. Astronauts with low immunity during spaceflight mission may be infected by these opportunistic pathogens. The development of a manned space program worldwide not only provides an opportunity to study the impact of this extreme environment on opportunistic pathogenic microbes, but also offers a unique platform to detect mutations in pathogenic bacteria. Various microorganisms have been carried on a spacecraft for academic purposes, including normal flora distributed in nature and in the human body, pathogenic bacteria, and opportunistic pathogens (Liu, 2017). Spaceflight influences the characteristics of these microorganisms (Higginson et al., 2016). *Acinetobacter baumannii* is a common drug‐resistant bacterium that causes persistent infections in hospitals, particularly in patients with low immunity. This study may provide better understanding of the mechanism of drug resistance from a clinical perspective. Space environment markedly influenced the SS1 strain. Some phenotypic, genomic, and transcriptomic changes were observed between the SS1 and GS1 strains.

In our study, the reference Aba strain was shown to express some genes associated with resistance to beta‐lactams, cephalosporin, fluoroquinolone, macrolides, aminoglycosides, and tetracycline. Drug resistance in GS1 and SS1 strains remained consistent in disk diffusion tests, and the drug resistance‐associated genes in the original Aba strain remained unchanged, suggesting that the *A. baumannii* strain maintained its drug‐resistant phenotype with regard to these antibiotics while grown during spaceflight. Hensley (2010) reported similar results, showing that clinical isolates of *A. baumannii* exhibited a resistant phenotype after a low‐sheer modeled microgravity experiment. Eight genes related to penicillin‐binding protein were also upregulated, and 11 genes related to beta‐lactamase were downregulated, thereby contributing to sulbactam resistance (Fernandez‐Cuenca et al., 2003; Krizova, Poirel, Nordmann, & Nemec, 2013; Penwell et al., 2015; Urban, Go, Mariano, & Rahal, 1995). Additionally, the *rpoB* gene was reportedly upregulated, thereby potentially contributing to rifampicin resistance (Giannouli et al., 2012; Houang, Chu, Lo, Chu, & Cheng, 2003). Furthermore, members of the *armA* gene family were upregulated (Liou, Yoshizawa, Courvalin, & Galimand, 2006), thereby potentially contributing to aminoglycoside resistance. One gene associated with multidrug resistance and with efflux pumps (*adeb*) was upregulated, whereas another gene with the same function was downregulated (Ruzin, Keeney, & Bradford, 2007). Two genes related to *BaeSR*, which regulate the resistance of efflux pumps, were downregulated (Lin, Lin, Yeh, & Lan, 2014). These changes in gene expression related to antibiotic resistance may influence the resistant phenotype of SS1 strain. However, none of these changes in gene expression were reflected in the resistant phenotype.

The flight strain SS1 exhibited the least intercellular mucus and the smoothest cell walls upon SEM imaging, suggesting that genes associated with the bacterial cell membrane, invasiveness, and adherence could be differently regulated in the SS1 strain compared with those in the GS1 strain. Outer membrane proteins constitute an important component of the bacterial cell membrane, including the lipopolysaccharide. Five genes associated with OmpA were downregulated; these normally play a key role in adhesion. Li et al. (2014) reported the same elongated cells in *K. pneumoniae* after spaceflight. Furthermore, Pyle et al. (1999) reported elongated *Burkholderia cepacia* cells and chains of cells after culture during spaceflight. Thus, these phenotypic changes could be a stress‐related or an adaptation reaction of microbiomes exposed to a microgravity environment.

Compared with the control strain GS1, the flight strain SS1 had significantly decelerated growth and decreased biofilm formation ability. Previous studies on *E. coli* and *B. subtilis* exposed to a microgravity environment reported different growth rates because of the differences in experimental conditions, including microgravity simulator, medium, growth stage, and experiment strain character, such as motile and nonmotile strains. (Brown, Klaus, & Todd, 2002; Kacena & Todd, 1997; Kacena, Leonard, Todd, & Luttges, 1997; Kacena, Manfredi, & Todd, 1999; Kacena, Merrell, et al., 1999; Klaus, Todd, & Schatz, 1998). Importantly, the growth rate of the flight strain SS1 was decreased, and this effect may be associated with the downregulation of genes involved in amino acid transport, metabolism, translation, ribosomal structure, and biogenesis. The same changes in growth rate were observed previously (Fajardo‐Cavazos & Nicholson, 2016; Su et al., 2014). Moreover, in the Space Environment Survivability of Living Organisms experiment, Nicholson et al. (2011) analyzed the survival and growth rates of *B. subtilis* spores on the O/OREOS spacecraft; compared with ground control experiments, the flight group of *B. subtilis* cells also showed lower growth rates.

We confirmed that the *csuA/B/D* genes were downregulated. Pilus biosynthesis is important during the initial biofilm formation period, and this process depends on the csuA/BABCDE system (Gaddy & Actis, 2009; Longo, Vuotto, & Donelli, 2014). Biofilm formation in *A. baumannii* requires expression of biofilm‐related genes, such as *csuA/B/D*. Additionally, changes in the biofilm formation ability are probably associated with changes in growth rates (Farshadzadeh et al., 2018). Zea et al. (2017) reported that *E. coli* cells in space accounted for 37% of the volume of the control group. Additionally, Mauclaire and Egli (2010) reported that two ISS isolates (*Micrococcus luteus* strains LT110 and LT100) produced significantly fewer extracellular polysaccharides under simulated microgravity, similar to that observed in a low shear stress environment, compared with normal gravity. Additionally, they predicted that this phenomenon may influence biofilm thickness and stability. We previously reported that genes associated with lipid biosynthesis were downregulated in strains cultured during spaceflight compared with those in the ground control strain, thereby potentially resulting in decreased cell membrane synthesis (Su et al., 2014). Roy et al. (2016) considered that the downregulation of the chemotaxis and flagellar machinery genes, ribosome genes, purine and pyrimidine metabolism, pentose phosphate pathway, glycolysis and fatty acid biosynthesis, LPS synthesis, and DNA replication may related with the higher survival rate in early hours. Genes *pga*B/C/D, which encode poly‐β‐1,6‐*N*‐acetylglucosamine—the major component of the biofilm exopolysaccharidic matrix—were downregulated. Herein, nine of the downregulated genes in the VFDB annotation system were related to the virulence factor type IV pili, which is associated with biofilm formation. The length of type IV pili is dynamic. They elongate via polymerization and retract via depolymerization. When the pilus adheres to an object during retraction, remarkably high force is applied on this object (Maier, Potter, So, Seifert, & Sheetz, 2002; Merz, So, & Sheetz, 2000). Additionally, two downregulated genes were associated with the virulence factor alginate, which contributes to biofilm formation, and two other genes were related to the virulence factor fimbriae, which contributes to adherence. The biofilm formation ability as well as the adherence ability of the SS1 strain was decreased after spaceflight, which correspond to the downregulation of *csuA/B/D*,* pga*B/C/Ds, and type IV pili‐related genes.

Comparative genomic analysis of SS1 and GS1 strains yielded only a few mutations compared with the reference Aba strain. Only one SNP was identified in the flight strain, and the function of this gene is still unknown. Most of the mutations were common between the GS1 and SS1 strains, suggesting that these strains underwent the same genomic changes, even when cultured under different environments and on exposure to different stressors.

Certain previous studies have reported the effect of spaceflight environment at the level of gene expression. *Salmonella typhimurium* was carried by space shuttle STS‐115, and Wilson et al. (2007) reported that *S. typhimurium* displayed increased virulence and extracellular matrix accumulation after spaceflight, consistent with a biofilm, upon SEM imaging and in the murine infection model, they observed an enhancement in the virulence of the flight strain. RNA‐binding protein Hfq caused some changes in gene expression, including biofilm formation and iron levels. Arunasri et al. (2013) found that under simulated microgravity conditions, 100 genes were significantly differently expressed in *E. coli*. Genes related to adaptation to stress, including those related to DNA replication and nucleoside metabolism, were upregulated. While genes associated with membrane transporters, such as ompC and exbB, were downregulated. Furthermore, genes related to carbohydrate catabolic processes were also downregulated. Crabbé et al. (2013) reported genes involved in flocculation regulatory pathways, which were differentially regulated in *C. albicans* after spaceflight, which may cause *C. albicans* aggregation and enhanced budding. Moreover, genes involved in biofilm formation and filamentation in *C. albicans* were also differentially expressed in the spaceflight‐cultured *C. albicans*. Comparative transcriptomic analysis revealed that the spaceflight strain SS1 displayed a significantly different gene expression profile compared with the ground control strain GS1. Changes in gene expression in the SS1 strain were associated with bacterial metabolism. In COG categories, many DEGs were classified into amino acid transport, metabolism, translation, ribosomal structure, and biogenesis. Analysis of GO categories revealed that DEGs between SS1 and GS1 were enriched in cellular components, molecular functions, and biological processes. Some categories were associated with formation of the membrane, location, and transport. Most DEGs enriched in these categories were downregulated. Only one KEGG pathway, associated with membrane receptor proteins containing iron complexes, was meaningful and also downregulated. Considering the functions of these DEGs, we considered numerous biological activities such as metabolism and transmembrane transporter, which were decreased in the SS1 strain. These differences between the SS1 and GS1 strains suggest that *A. baumannii* adapt to stress and survive in extreme environments, such as a spacecraft, via regulation of important metabolic functions and biogenesis pathways.

In the MARS 500 project, opportunistic pathogens and stress‐tolerant bacteria were thought to develop multi‐stage, ground‐based simulation experiment. Furthermore, bacterial diversity decreased with time. Opportunistic pathogens with high abundance and biofilm formation ability were detected in both residential and functional environments and under stress conditions (Schwendner et al., 2017). However, our study is the first to analyze the mechanism underlying the survival of *A. baumannii* and the alterations they undergo upon culturing during spaceflight at the phenotypic, genetic, and transcriptomic levels.

As in most previous spaceflight experiments, the design of the Shenzhou 11 spacecraft did not allow for measurement of growth and drug resistance during spaceflight. Therefore, it was not possible to determine growth rates in space. This limitation may be resolved in the 2020s after the launch of China's new space station, which will have a medical sample analysis cabinet at its core center. Nonetheless, this study shows various changes in the phenotypic, genomic, and transcriptomic characteristics of the flight strain SS1 compared with the ground control strain GS1 after 33 days of culturing in the Shenzhou 11 spacecraft. The environment in the spacecraft after 33 days of spaceflight is complex, involving short‐time overweight, long‐time microgravity, vibration, and extremely high and low temperatures. We predict that longer spaceflight times may provide better insights into the effects of these conditions on pathogenic bacteria such as *A. baumannii*.

## 5 CONCLUSIONS

China is preparing to launch a new space station to house astronauts for 180 d or longer. Further studies are needed to determine the effects of longer spaceflight times on bacteria (Liu, 2017). In the current study, we explored changes in phenotypic, genomic, and transcriptomic characteristics of multidrug‐resistant *A. baumannii* after culturing on the Shenzhou 11 spacecraft for 33 days of spaceflight. Expression of genes associated with bacterial biofilm formation ability, metabolism, and drug resistance was altered after spaceflight. Prospective studies examining the effect of spaceflight on drug‐resistant pathogenic bacteria are essential for microbiological surveillance of space stations and are expected to have beneficial effects on the health of future astronauts during long‐term space travel.

## CONFLICT OF INTEREST

None declared.

## AUTHORS CONTRIBUTION

Z.X. and Y. Y. contributed equally to this paper. C.L designed and coordinated the project. Z.X., Y.Y., Z.X.L., H.B., X.C., L.D.G, B.P., and Z.B. performed laboratory experiments. Z.X. and Y.Y. performed the data analysis. C.L. wrote the manuscript with assistance from all authors. All authors read and approved the final manuscript.

## ETHICS STATEMENT

This article does not contain any studies with human or animals performed by any of the authors.

## Data Availability

All data have been uploaded to GenBank. The accession numbers of the complete genome of Aba strain are CP030083 and CP030084. Accession numbers for resequencing data of the SS1 and GS1 strains are SAMN09453122 and SAMN09453123, respectively. Accession numbers for transcriptomic data of the SS1 and GS1 strains are SAMN09453347 and SAMN09453348, respectively.
